# Patient Eligibility for Transoral Endocrine Surgery Procedures in the United States

**DOI:** 10.1001/jamanetworkopen.2019.4829

**Published:** 2019-05-31

**Authors:** Raymon H. Grogan, Insoo Suh, Kate Chomsky-Higgins, Salman Alsafran, Elya Vasiliou, Christopher R. Razavi, Lena W. Chen, Ralph P. Tufano, Quan-Yang Duh, Peter Angelos, Jonathon O. Russell

**Affiliations:** 1 Endocrine Surgery Program, Michael E. DeBakey Department of Surgery, Baylor College of Medicine, Houston, Texas; 2 Section of Endocrine Surgery, Department of Surgery, University of California, San Francisco; 3 Endocrine Surgery Program, Department of Surgery, University of Chicago, Chicago, Illinois; 4 Division of Head and Neck Endocrine Surgery, Department of Otolaryngology–Head and Neck Surgery, Johns Hopkins University, Baltimore, Maryland

## Abstract

**Importance:**

Transoral endocrine surgery (TES) allows thyroid and parathyroid operations to be performed without leaving any visible scar on the body. Controversy regarding the value of TES remains, in part owing to the common belief that TES is only applicable to a small, select group of patients. Knowledge of the overall applicability of these procedures is essential to understand the operation, as well as to decide the amount of effort and resources that should be allocated to further study the safety, efficacy, and value of these operations.

**Objective:**

To determine what percentage of US patients undergoing thyroid and parathyroid surgery are eligible for TES using currently accepted exclusion criteria.

**Design, Setting, and Participants:**

Cross-sectional study of 1000 consecutive thyroid and parathyroid operations (with or without neck dissection) performed between July 1, 2015, and July 1, 2018, at 3 high-volume academic US thyroid- and parathyroid-focused surgical practices (2 general surgery, 1 otolaryngology–head and neck endocrine surgery). Eligibility for TES was determined by retrospectively applying previously published exclusion criteria to the cases.

**Main Outcomes and Measures:**

The primary outcome was the percentage of thyroid and parathyroid cases eligible for TES. Secondary outcomes were a subgroup analysis of the percentage of specific types of cases eligible and the reasons for ineligibility.

**Results:**

The mean (SD) age of the 1000 surgical patients was 53 (15) years, mean (SD) body mass index (calculated as weight in kilograms divided by height in meters squared) was 29 (7), and 747 (75.0%) of the patients were women. Five hundred fifty-eight (55.8%) of the patients were eligible for TES. Most patients with thyroid nodules with cytologically indeterminate behavior (165 of 217 [76.0%]), benign thyroid conditions (166 of 240 [69.2%]), and primary hyperparathyroidism (158 of 273 [57.9%]) were eligible for TES, but only 67 of 231 (29.0%) of patients with thyroid cancer were eligible. Among all 1000 cases reviewed, previous neck operation (97 of 441 [22.0%]), nonlocalized primary hyperparathyroidism (78 of 441 [17.7%]), and need for neck dissection (66 of 441 [15.0%]) were the most common reasons for ineligibility.

**Conclusions and Relevance:**

More than half of all patients undergoing thyroid and parathyroid surgery in this study were eligible for TES. This broad applicability suggests that a prospective multicenter trial is reasonable to definitively study the safety, outcomes, and cost of TES.

## Key Points

**Question:**

What proportion of patients who present for thyroid or parathyroid surgery are eligible for a scarless transoral operation based on currently accepted exclusion criteria?

**Findings:**

In this cross-sectional study of 1000 surgical patients across 3 US academic medical centers, 558 patients (55.8%) who underwent thyroid or parathyroid surgery would have been eligible for a scarless transoral approach.

**Meaning:**

Transoral endocrine surgical procedures are more broadly applicable to patients undergoing thyroid and parathyroid surgery than previously thought, and definitive studies on the safety, efficacy, and cost of the transoral approach are warranted.

## Teaser

This cross-sectional study evaluated 1000 thyroid and parathyroid operations performed at 3 US academic medical centers to assess the percentage of these operations that could be performed using transoral endocrine surgery, an approach that leaves no external scarring.

## Introduction

Transoral endocrine surgery (TES) comprises a group of operations that allow thyroidectomies and parathyroidectomies to be done without leaving a visible scar on the patient’s neck. As the name implies, access to the neck is via the oral cavity. Currently, the main advantage of these operations is the lack of a visible scar in an area that is not easily hidden. The most common TES techniques currently used in the United States are TOETVA (transoral endoscopic thyroidectomy vestibular approach) for thyroidectomy and TOEPVA (transoral endoscopic parathyroidectomy vestibular approach) for parathyroidectomy. The transoral vestibular approach was first described in 2011 in cadavers by Richmon et al^1^ and later popularized by Anuwong^2^ in humans. This approach has supplanted all other oral access approaches^3^ to the thyroid and parathyroid because of its improved safety and feasibility; thus, TES may also be referred to as TOVAES (transoral vestibular approach endocrine surgery). Proponents of TES cite the proximity to the target anatomy as one of the main advantages of these operations over other minimally invasive or remote-access approaches to the neck. In addition, because the aggregate length of these incisions is typically 2 cm or less, TES could be considered a minimally invasive procedure for thyroidectomy, and either a minimally invasive or a remote-access procedure for parathyroidectomy. The first endoscopic and robotic TES operations were performed in the United States in April 2016.^4,5^ It is estimated that as of January 2019 approximately 300 of these procedures have been performed in the United States, across multiple centers.

A series of 425 cases by Anuwong et al^6^ is the largest series of TES cases published to date. In this series, TES outcomes were compared with equivalent open procedures using propensity score matching. The authors concluded that the TES approach was equivalent to open technique in terms of safety, with similar rates of recurrent laryngeal nerve injury and hypoparathyroidism. In addition, no wound infections and no additional permanent complications were associated with this approach. In spite of these published data, there remains significant skepticism in the United States in regard to safety and outcomes. In addition, there has been debate regarding how widely applicable these procedures are in the general population, with some authors suggesting that only a very small portion of thyroid and parathyroid operations can be done using these techniques.^7^ To definitively prove the safety and outcomes of TES, a multicenter clinical trial will be necessary. However, such a study would not answer the question as to how widely applicable TES might be to the broader set of patients undergoing endocrine surgery. Critics of TES claim that it has limited applicability owing to extensive exclusion criteria.^8^ If the approach can only be used in a limited number of thyroid and parathyroid surgery candidates, its value is questionable. Understanding how widely applicable these procedures are to this patient population would be helpful to determine how much time and resources should be put into a large multicenter trial to completely define the safety profile of TES. In addition, such studies would help our understanding of what the future of TES might look like in the United States. Despite some claims in the literature, it is currently unknown how widely applicable TES is because, to our knowledge, there are no studies that attempt to answer this question.

Given the above information, we set out to better understand how broad or narrow the scope of TES is in the United States. We retrospectively reviewed a consecutive series of 1000 surgical cases from 3 different academic endocrine surgery practices in the United States. We then applied a standard set of TES inclusion and exclusion criteria to these cases to determine how many patients who presented for an operation would have been eligible for these procedures in an US academic thyroid and parathyroid surgery practice.

## Methods

This study followed the Strengthening the Reporting of Observational Studies in Epidemiology (STROBE) reporting guideline. A cross-sectional study of consecutive operations performed in 3 US academic thyroid and parathyroid surgery practices (University of Chicago, Johns Hopkins University, and the University of California, San Francisco) between July 1, 2015, and July 1, 2018, was performed. The study was reviewed and approved by the institutional review boards at all 3 academic institutions. Owing to the deidentified and retrospective nature of the data analysis, all 3 institutional review boards waived informed consent.

One thousand consecutive cases of parathyroidectomy and thyroidectomy with or without central and lateral neck lymph node dissections performed during the study period at the 3 institutions combined were included for analysis. An equal number of cases were reviewed for each institution. The time required to accrue the cases varied between the 3 institutions based on their respective volumes; however, all cases presented to the 3 institutions within the above study period. Patient demographics, indication for operation, and type of operation were recorded and analyzed. To determine how many cases would have been eligible for TES, we applied a standard set of exclusion criteria as previously described in the literature.^9,10,11^ These exclusion criteria were serious medical comorbidity that would contraindicate a prolonged procedure time; previous external beam radiation to the neck; previous open neck operation; benign single nodule larger than 6 cm; malignant thyroid tumor larger than 2 cm; total thyroid lobe diameter larger than 10 cm; substernal thyroid; indications for a therapeutic central neck dissection; indications for a lateral neck dissection; nonlocalized primary hyperparathyroidism; secondary hyperparathyroidism; and tertiary hyperparathyroidism. Any patient who met 1 or more of these exclusion criteria was deemed ineligible for TES. After applying the exclusion criteria, the cases were then tabulated to determine the percent eligible rate.

### Statistical Analysis

Analysis of variance was used for multiple group comparisons of means, and the χ^2^ test was used for categorical variable analysis. All statistical analysis was done using the Stata SE15 statistical package (StataCorp), and *P* < .05 (2-sided) was considered statistically significant.

## Results

The study included 1000 thyroid and parathyroid surgery procedures. Of the 1000 surgical patients in the cases evaluated, the mean (SD) age was 53 (15) years, 747 (75.0%) of the patients were women, and the mean (SD) body mass index (calculated as weight in kilograms divided by height in meters squared) was 29 (7) (Table 1). The mean (SD) thyroid lobe volume and the mean (SD) thyroid nodule size were smaller for cancer than for benign thyroid conditions (40 [72] mL benign volume vs 12 [16] mL cancer volume; 3.3 [1.8] cm benign nodule size vs 2.2 [1.6] cm cancerous nodule size). Combining the operative indications into generalized thyroid and parathyroid disease shows that among the 1000 cases, the most common diagnosis for operation was a benign thyroid condition or a cytologically indeterminate behavior thyroid nodule (457 [45.7%]) followed by primary hyperparathyroidism (273 [27.3%]), thyroid cancer (231 [23.1%]), and then secondary or tertiary hyperparathyroidism (37 [3.7%]) (Table 2). The 3 most common types of operations performed were total thyroidectomy (375 of 1000 [37.5%]), thyroid lobectomy (287 of 1000 [28.7%]) and focused parathyroidectomy (244 of 1000 [24.4%]). The complete list of patient demographic characteristics and indications for operation are shown in Table 1 and Table 2.

**Table 1.** Demographic Characteristics of Study Cohort

Characteristic	Mean (SD)				*P* Value
	Institution 1 (n = 334)	Institution 2 (n = 333)	Institution 3 (n = 333)	Total Patients (N = 1000)	
Demographic					
Age, y	54 (15)	54 (15)	50 (15)	53 (15)	<.001
Female sex, No. (%)	258 (77.3)	243 (73.0)	246 (73.9)	747 (75.0)	.41
BMI	30 (8)	28 (6)	29 (7)	29 (7)	.001
Benign thyroid					
Lobe volume, mL	52 (104)	30 (36)	38 (56)	40 (72)	<.001
Nodule size, cm	3 (2)	3.3 (1.8)	3.5 (1.6)	3.3 (1.8)	.001
Thyroid cancer					
Lobe volume, mL	9 (11)	12 (16)	15 (19)	12 (16)	<.001
Nodule size, cm	1.6 (1)	2.3 (1.9)	2.7 (1.6)	2.2 (1.6)	<.001

Abbreviation: BMI, body mass index, calculated as weight in kilograms divided by height in meters squared.

**Table 2.** Indications for Operation

Indication for Operation	No. (%)				*P* Value
	Institution 1 (n = 334)	Institution 2 (n = 333)	Institution 3 (n = 333)	Total Patients (N = 1000)	
1° Hyperparathyroidism	121 (36.2)	107 (32.1)	45 (13.5)	273 (27.3)	<.001
Benign thyroid condition	55 (16.5)	59 (17.7)	126 (37.9)	240 (24.0)	<.001
Thyroid cancer	62 (18.6)	84 (25.2)	85 (25.5)	231 (23.1)	.05
Indeterminate thyroid nodule	72 (21.6)	71 (21.3)	74 (22.2)	217 (21.7)	.96
2° or 3° hyperparathyroidism	23 (6.9)	12 (3.6)	2 (0.6)	37 (3.7)	<.001
Other^a^	1 (0.2)	0 (0)	1 (0.3)	2 (0.2)	.61

^a^ Other includes prophylactic thyroidectomy for medullary thyroid cancer and lingual thyroid.

### Interinstitutional Comparisons

With the exception of sex, all other demographic features, tumor sizes, and thyroid volumes being operated on differed among the institutions. Similarly, the types of operations being performed and the indications for operation by disease group were all different among the institutions. For example, institution 1 had a lower percentage of operations performed for thyroid cancer (62 of 334 [18.6%]) compared with institutions 2 (84 of 333 [25.2%]) and 3 (85 of 333 [25.5%]). Similarly, institution 2 had a greater percentage of thyroid lobectomies (126 of 333 [37.8%]) compared with institutions 1 (75 of 334 [22.5%]) and 3 (86 of 333 [25.8%]). Although each of these institutions adheres to the most recent American Thyroid Association guidelines for the management of thyroid disease, practice patterns vary.^12^ Overall, the 3 academic institutions represent 3 different patient populations across different surgical training backgrounds, with different disease prevalence, and, by extension, different types of operations performed.

### The TES-Eligible Population

We applied the exclusion criteria to the consecutive cases studied at the 3 institutions. With all cases combined among the 3 institutions, 558 of 1000 (55.8 %) of patients who underwent thyroid and parathyroid surgery in these academic practices were eligible for TES. The differences in disease presentation among the institutions also resulted in the percentage eligible for TES being different among the 3 institutions (51.2%, 50.8%, and 65.5%; *P* < .001) (Table 3). The majority of patients with cytologically indeterminate behavior thyroid nodules (165 of 217 [76.0 %]) and benign thyroid conditions (166 of 240 [69.2%]) were eligible for TES. Likewise, a majority of patients with primary hyperparathyroidism were also eligible for TES (158 of 273 [57.9%]). In contrast, 67 of 231 (29.0%) of patients with thyroid cancers were eligible (Table 3). The most common reasons for not being eligible for TES (Table 4) were (1) having a reoperation after a neck incision had already been made in a previous operation (97 of 441 [22.0%]); (2) lack of preoperative localization in cases of primary hyperparathyroidism (78 of 441 [17.7%]); (3) the need for a central or lateral neck dissection (66 of 441 [15.0%]); (4) multiple contraindications (62 of 441 [14.1%], see Table 5 for details); and (5) differentiated thyroid cancerous tumor larger than 2 cm (53 of 441 [12.0%]).

**Table 3.** Percentage of Patients Eligible for TES by Diagnosis

Eligible for TES	No./Total No. (%)^a^				*P* Value
	Institution 1 (n = 334)	Institution 2 (n = 333)	Institution 3 (n = 333)	Total Patients (N=1000)	
Indeterminate thyroid nodule	58/72 (80.6)	43/71 (60.6)	64/74 (86.5)	165/217 (76.0)	.08
Benign thyroid condition	36/55 (65.5)	34/59 (57.6)	96/126 (76.2)	166/240 (69.2)	<.001
1° Hyperparathyroidism	54/121 (44.6)	68/107 (63.6)	36/45 (80.0)	158/273 (57.9)	<.001
Thyroid cancer	22/62 (35.5)	24/84 (28.6)	21/85 (24.7)	67/231 (29.0)	.89
Other^b^	1/1 (100)	0/0	0/0	1/2 (50.0)	.37
Total	171/334 (51.2)	169/333 (50.8)	218/ 333 (65.5)	558/1000 (55.8)	<.001

Abbreviation: TES, transoral endocrine surgery.

^a^ Denominators are from indications for operation from each diagnosis as shown in Table 2.

^b^ Other includes prophylactic thyroidectomy for medullary thyroid cancer and lingual thyroid.

**Table 4.** Indications for Ineligibility for TES

Reason Not Eligible for TES	No. (%)^a^				*P* Value
	Institution 1	Institution 2	Institution 3	Total Patients	
Reoperation	28 (17.2)	46 (28.1)	23 (20.2)	97 (22.0)	<.001
Nonlocalized 1° hyperparathyroidism	52 (31.9)	17 (10.4)	9 (7.9)	78 (17.7)	<.001
Neck dissection for cancer	16 (9.8)	15 (9.2)	35 (30.7)	66 (15.0)	<.001
Multiple contraindications	18 (11.0)	31 (18.9)	13 (11.4)	62 (14.1)	.01
Cancerous tumor >2 cm	16 (9.8)	23 (14.0)	14 (12.3)	53 (12.0)	.26
2° or 3° Hyperparathyroidism	21 (12.9)	3 (1.8)	1 (0.9)	25 (5.7)	<.001
Medical comorbidities	3 (1.8)	16 (9.8)	5 (4.4)	24 (5.4)	<.001
Benign nodule >6 cm	6 (3.7)	7 (4.3)	2 (1.8)	15 (3.4)	.24
Substernal goiter	3 (1.8)	6 (3,7)	1 (0.9)	10 (2.3)	.15
Thyroid lobe >10 cm	0	0	8 (7.0)	8 (1.8)	<.001
Previous neck radiation	0	0	2 (1.8)	2 (0.45)	.13
Lingual thyroid	0	0	1 (0.9)^b^	1 (0.23)	.37
Total	163 of 334 (48.8)	164 of 133 (49.3)	114 of 333 (34.2)	441 of 1000 (44.1)	<.001

Abbreviation: TES, transoral endocrine surgery.

^a^ Percentage of all operations not eligible for TES.

**Table 5.** Breakdown of Cases With Multiple Contraindications for Transoral Endocrine Surgery^a^

Multiple Contraindications	No. (%)				*P* Value
	Institution 1	Institution 2	Institution 3	Total Patients	
Neck dissection for cancer	8 (19.0)	18 (24.3)	12 (38.7)	38 (25.8)	.12
Cancer >2 cm	11 (26.2)	15 (20.3)	12 (38.7)	38 (25.8)	.70
Nonlocalized 1° HPT	0 (0)	13 (17.6)	0 (0)	13 (8.8)	<.001
Medical comorbidities	1 (2.4)	10 (13.5)	1 (3.2)	12 (8.2)	.001
2° or 3° hyperparathyroidism	1 (2.4)	9 (12.2)	0 (0)	10 (6.8)	.001
Benign nodule >6 cm	7 (16.6)	2 (2.7)	1 (3.2)	10 (6.8)	.04
Substernal goiter	6 (14.3)	6 (8.1)	1 (3.2)	13 (8.8)	.14
Thyroid lobe >10 cm	8 (19.0)	1 (1.3)	1 (3.2)	10 (6.8)	.007
Previous neck radiation	0	0	1 (3.2)	1 (0.7)	.37
Anaplastic	0	0	1 (3.2)	1 (0.7)	.37
Thyroid lymphoma	0	0	1 (3.2)	1 (0.7)	.37
Total	42 (100)	74 (100)	31 (100)	147 (100)	<.001

Abbreviation: HPT, hyperparathyroidism.

^a^ This is a breakdown of cases listed in Table 4.

## Discussion

Herein for the first time, to our knowledge, we demonstrate the potential, based on our results, that 55.8% (range, 50.8%-65.5%) of patients who present to an academic thyroid and parathyroid surgery practice in the United States are eligible for TES. The 3 institutions represented both general surgery (2 institutions) and otolaryngology–head and neck surgery–trained disciplines (1 institution), and substantial variations in disease presentation are represented among the practices. Given this variation and representation of both subspecialties, we believe these data are broadly applicable to all patients undergoing thyroid and parathyroid operations in academic medical centers. It is estimated that 150 000 thyroidectomies and 100 000 parathyroidectomies are performed annually in the United States.^13,14,15^ Applying a 56% eligibility shows that it is possible that as many as 140 000 patients per year in the United States could be eligible for TES. Considering that up to 140 000 thyroid and parathyroid operations could be performed via TES annually in the United States, our results suggest that TES is applicable to a large number of patients and may not be considered a “boutique” operation in the near future.

The adoption and expansion of TES depend on several factors. First and foremost, it must be proven to be at least as safe as the open surgical approach and to have equivalent outcomes. The case series by Anuwong and colleagues^6^ is helpful in this regard and clearly demonstrates that infection is not an issue in the hands of experienced surgeons. In 425 procedures, there were no infections. This is clear evidence that infection rates are not as concerning as initially feared and is consistent with the anecdotal experience of the authors. However, the case series by Anuwong and colleagues^6^ remains that of a single insitution; thus, it remains unclear whether the low rate of infection, recurrent laryngeal nerve injury, and hypoparathyroidism rates can be replicated by other surgeons. Early reports from the United States are promising, however.^16^ In addition, the incidence of other technique-specific complications remains unknown. The only way to prove that other surgeons can in fact replicate the success shown in the study by Anuwong et al^6^ is with a multicenter, multisurgeon trial. While these data are accumulating, training and adoption of the new technique should be done in a safe and thoughtful manner.^17^ Second, it must be demonstrated whether the abscence of a cutaneous incision either improves quality of life or adds value for patients. Again, data are limited in this regard.^18,19,20,21^ Given that the present study shows that hundreds of thousands of patients with thyroid and parathyroid disease could be treated with TES in the United States, we believe our findings strongly support the need for definitive studies on safety and efficacy. Other questions regarding the procedure, such as cost, physician perceptions, and patient-centered outcomes, could also be answered by a trial.

Although incision size is not the only criterion that classifies an operation as minimally invasive, some consider the traditional approach to focused parathyroidectomies to already be minimally invasive, if it is performed with a 2-cm incision.^22^ If that is the case, the merits of moving that 2-cm incision from the neck to the inside of the lower lip may be questioned by some. However, it is not clear how many surgeons actually perform a “minimally invasive” parathyroidectomy via a 2-cm incision. In addition, recent advances in TES parathyroidectomy have reduced the total incision size to 1 cm in select cases through the use of pediatric laparoscopic trocars. If the operation is as safe as the traditional approach, we believe the preference of avoiding a cervical scar should be left to individual patients as long as they are eligible for TES. The patient's autonomy in choosing TES is especially relevant because it has been demonstrated that patients are concerned about cervical scars no matter how well they heal or how small they are.^23^ However, even if the parathyroidectomies are removed from the indications for TES in this population, there are still more than 80 000 patients undergoing thyroidectomies done each year in the United States who are potentially eligible for this scarless technique, thus justifying further exploration of this approach.

As stated, the value of not having a scar on the neck has been questioned. However, data continue to accumulate showing that scars, particularly in very prominent locations such as the front of the neck, are bothersome to some people, and if given a choice many would prefer to not have that scar visible.^18,24^ Previous US studies have concluded that more than 10% of patients undergoing thyroidectomy consider a scar revision even years after surgery, and that 50% of patients are “extremely satisfied” with their scar.^21^ Preliminary data from Johns Hopkins University have shown that there is a penalty in overall attractiveness for patients with a Kocher incision, and that patients would be willing to pay more than $10 000 to avoid this incision.^24^ In that same study, the authors demonstrated that, in patients who underwent a TES procedure, there was no attractiveness penalty. Although the value of avoiding a cervical incision remains to be fully studied, it is apparent that at least some patients or potential patients prefer to avoid a cervical incision, even if there is additional surgical risk or cost.^24^

### Limitations

One of the main limitations of this study is that the predictive model only applies to the demographic characteristics of patients of an academic thyroid and parathyroid surgery practice. This particular patient population is likely not representative of the larger population of patients who undergo endocrine surgery in the United States, because most thyroidectomies are still done in private practice settings. However, it is likely that our predictions are an underestimate of the overall population of patients who undergo endocrine surgery in the United States, because presumably an academic thyroid and parathyroid surgery practice has a greater proportion of cases of reoperations, neck dissections, nonlocalized parathyroids, and advanced-stage cancers that would not be eligible for TES. Thus, it is likely that more than 56% of the general population of patients treated for thyroid and parathyroid disease is eligible for TES. However, even if this exact percentage is not generalizable to the entire population, it remains true that a significant number of these operations are eligible for TES in the United States. Our data are also limited because we did not exclude patients who may have been ineligible for TES because of an anatomic reason. Examples of this would be active oral or dental infections, presence of mandibular implants, anatomic defects of the mandible, or cancers of the oral cavity. In the authors’ anecdotal experience, these issues are relatively rare in patients undergoing thyroid and parathyroid surgery; however, they may slightly lower the overall percentage of the population eligible for TES. One important factor not considered herein is patient preference. Our data only show those patients who are technically eligible; therefore, we have no way of knowing at this time how many patients would actually prefer a scarless alternative such as TES. It is likely that in the United States, the patient’s perception of TES is highly associated with safety as well as the opinions of American surgeons and other clinicians. The stigma of a neck scar may not be as significant to patients in the United States relative to some other countries, which could affect adoption and uptake rates, particularly in the early adoption phase of TES. Therefore, if safety and outcomes are proven similar, and surgeons develop familiarity and facility with the procedures, it is possible that uptake rates among the US public will increase as well. Finally, our paper does not address other outstanding questions and concerns regarding TES, such as cost, physician perceptions of the operation, patient perceptions of the operation, and strategies for safe training and broad adoption of the technique. We believe many of these issues could and should be addressed with a clinical trial before widespread adoption of the technique.

## Conclusions

Our data demonstrated that TES was applicable to more than half of all open thyroid and parathyroid surgery candidates in academic practices included in this study when standard inclusion and exclusion criteria were applied. Results of this tudy suggest that there are potentially hundreds of thousands of patients who may be eligible to forego a cutaneous incision. We believe these data show that TES is not and should not be considered an operation with limited applicability. Transoral endocrine surgery has the potential to improve the lives of a large number of patients, and thus it should be considered a viable option in the United States. Based on these data, a large, prospective multicenter trial is warranted to further evaluate the safety, clinical outcomes, patient-centered outcomes, and costs of TES.
